# Long‐Duration Response to Levodopa in the PPMI‐Cohort

**DOI:** 10.1002/mds.70344

**Published:** 2026-05-09

**Authors:** Nils Schnalke, Tim Feige, Tom Hähnel, Björn Falkenburger

**Affiliations:** ^1^ Department of Neurology, Medical Faculty and University Hospital Carl Gustav Carus TUD Dresden University of Technology Dresden Germany; ^2^ Center for Neurodegenerative Diseases within the Helmholtz Association (DZNE) Dresden Germany

**Keywords:** levodopa response, long duration, Parkinson's disease, PPMI, progression

## Abstract

**Background:**

Treatment of Parkinson's disease (PD) with levodopa results in a sustained reduction of symptoms. Although the plasma half‐life of levodopa is short, it elicits a lasting effect, the long‐duration levodopa response (LDR). A decrease in LDR as PD progresses has been linked to motor complications, but long‐term data on the LDR and its clinical implications remain scarce.

**Objectives:**

The aim is to analyze the magnitude and impact of the LDR over time using data from the Parkinson's Disease Progression Marker Initiative (PPMI).

**Methods:**

First, therapy‐naïve Movement Disorder Society‐Unified Parkinson's Disease Rating Scale part III (MDS‐UPDRS III) scores were predicted using a mixed linear model (MLM) from n = 245 untreated people with PD (PwPD). This model yielded an increase of MDS‐UPDRS III scores of 2.65 points per year. Using this model, we then calculated LDR and short‐duration response in longitudinal data of 148 initially therapy‐naïve PwPD. Symptom progression was analyzed using correlation analyses and MLMs.

**Results:**

In the 98 PwPD with observed LDR, the LDR accounted for approximately half of the total levodopa response. No significant change in LDR magnitude was observed over up to 10 years (analysis of variance, *P* = 0.14; generalized estimating equations, *P* = 0.26). The LDR magnitude was not associated with the onset of motor complications. PwPD with absent LDR (n = 50) progressed faster than PwPD with observed LDR in several motor and non‐motor domains.

**Conclusions:**

The LDR is a stable component of the levodopa response and needs to be considered in clinical trials. These findings argue against a declining LDR as a major driver of motor fluctuations in PD. © 2026 The Author(s). *Movement Disorders* published by Wiley Periodicals LLC on behalf of International Parkinson and Movement Disorder Society.

Parkinson's disease (PD) is characterized by significant heterogeneity in symptoms and progression.^1^ Several factors have been described to influence disease progression, including GBA1 heterozygosity^2^ and PD subtypes that are based on clinical symptoms in data‐driven approaches.^3^, ^4^, ^5^ Trials that modify disease progression are urgently needed, and efforts to demonstrate therapeutic effects via fluid biomarkers have largely failed.^6^, ^7^

Clinical studies investigating effects on disease progression, therefore, typically use standardized clinical scales such as the Movement Disorder Society‐Unified Parkinson's Disease Rating Scale part III (MDS‐UPDRS III), inferring disease modification from slower progression in the verum than in the placebo group.

Measuring disease modification via progression of motor symptoms using the MDS‐UPDRS III is hampered by several problems. First, PD progresses slowly, necessitating long follow‐up durations to obtain meaningful information about disease modification.^8^ Second, most disease modifying studies allow use of levodopa and other standard of care (SoC) medications during the trial, which obscures the progression of motor symptoms. To mitigate this problem, the MDS‐UPDRS III is generally recorded in a practically defined *off*‐state, which is typically defined as more than 12 hours after the last intake of levodopa or other dopaminergic medication.^9^, ^10^, ^11^ Whether this corresponds to a “true” *off*‐state seems questionable, as temporally differential effects of levodopa on PD symptoms have already been described in the 1960s.^12^ They are termed the short‐duration and the long‐duration levodopa response (SDR and LDR)—with the latter lasting days to weeks after discontinuation of levodopa.^13^ A recent study in drug‐naïve patients in Ghana found that the LDR is responsible for approximately 60% of the reduction in UPDRS III scores in comparison to pre‐drug baseline,^14^ with others reporting similar effect sizes.^8^, ^15^ If the LDR is unaccounted for, this significantly hampers the ability of disease‐modifying trials to detect differences between (1) different patients and (2) different time points in the same patients.^16^

To date, studies examining the development of the LDR in larger cohorts remain scarce and it has not yet been reliably quantified. Furthermore, it has not yet been fully elucidated how LDR relates to disease progression and the occurrence of dyskinesias. Specifically, it has remained controversial whether the LDR is lost^13^, ^17^ or preserved^14^ as PD progresses. Using the pragmatic definition of the LDR previously proposed,^14^ we, therefore, investigated the long‐term development of the LDR using publicly available data from the Parkinson's Progression Marker Initiative (PPMI).

## Methods

### Dataset

Publicly available data of de novo people with PD (PwPD) from the PPMI cohort (NCT04477785) were downloaded in November 2023. For patients on dopaminergic medication, the yearly PPMI follow‐up visits include at least one MDS‐UPDRS III assessment in the *off* condition and a second rating in the *on* condition. The PPMI dataset is publicly available, and the original study was performed in accordance with the Declaration of Helsinki. Written informed consent was obtained from all participants and each separate PPMI center received approval from the respective ethics committee.^18^

### Definition of SDR and LDR

Definitions of SDR and LDR were chosen to be consistent with previous studies.^8^, ^14^, ^15^ The SDR was defined as the difference in the MDS‐UPDRS III after the acute levodopa challenge compared with the MDS‐UPDRS III score before the acute levodopa challenge (MDS‐UPDRS III *off*–MDS‐UPDRS III *on*).

The LDR was defined as the differences between the MDS‐UPDRS III score in the practically defined *off* condition and the estimated MDS‐UPDRS III score “as if levodopa had never been initiated” (drug‐naïve).

A “measured LDR” was defined for the first study visit after initiation of levodopa as the difference between the last pre‐levodopa MDS‐UPDRS III score and the first practically defined MDS‐UPDRS *off* score after levodopa initiation (Fig. S1). This yields exactly one “measured” LDR per study participant.

For time points later than 1 year after levodopa initiation, a “model‐derived” LDR was defined as the difference between the “predicted naïve” MDS‐UPDRS III (see below) and the MDS‐UPDRS III scores obtained during the study visits in the practically defined *off* state (MDS‐UPDRS III *off*).

The total response (TR) to levodopa was defined as the sum of LDR and SDR, that is, as the difference between the “predicted naïve” MDS‐UPDRS III and the MDS‐UPDRS III *on*.

### Calculation of Predicted Drug‐Naïve MDS‐UPDRS III Scores

“Predicted naïve” MDS‐UPDRS III scores were obtained using a mixed linear model (MLM) based on untreated PPMI participants. The MLM included 245 PwPD from the PPMI cohort with at least three consecutive MDS‐UPDRS III assessments without any antiparkinsonian medication. We used a random intercept and a fixed slope, with the first MDS‐UPDRS III score of each participant included as a covariate. A linear model (as opposed to non‐linear models) was considered appropriate as motor disability has been shown to progress similarly to a linear function.^19^, ^20^ The model converged when three consecutive MDS‐UPDRS III scores were used. Including additional demographic variables did not improve the model fit. When using a random slope, the model did not converge. The slope parameter of this model using all available data of the 245 PwPD corresponded to an increase of 2.65 points/year on the MDS‐UPDRS III scale, which is in line with a previously published estimate of 2.4 points/year.^19^ Additionally, this is in line with a recently published scale‐independent long‐term progression estimate of 2%/year of the maximum disability (MDS‐UPDRS III maximum: 132 points, 2.65/132 = 0.02).^21^ As untreated motor progression might be steeper, this approach likely yields a conservative estimate of MDS‐UPDRS III progression over time and, consequently, a conservative estimate of LDR magnitude.^20^

### Simulation of a Clinical Trial

To demonstrate the effect of the LDR in the context of a clinical trial investigating disease‐modifying effects, we selected PPMI participants into a “trial cohort” using the following inclusion and exclusion criteria.

#### Inclusion Criteria

(1) We selected PwPD within 2 years after PD diagnosis without any PD medication at the baseline visit (as per PPMI inclusion criteria) and restricted our analysis to PwPD who had at least one levodopa challenge within 1.5 years after initiation of levodopa to improve comparability between *off* scores.

(2) An initial treatment with dopamine agonists (DAs) (ie, after baseline but before the start of levodopa) was allowed if it was subsequently discontinued and if the MDS‐UPDRS III *off* assessment was not conducted while DAs and levodopa were prescribed at the same time.

(3) To obtain a meaningful number of PwPD, SoC drugs other than DAs were allowed after baseline (ie, monoamine oxidase B [MAO‐B]‐inhibitors [selegiline, rasagiline, safinamide], amantadine and anticholinergic drugs).

#### Exclusion Criterion

(1) Participants in whom the change in MDS‐UPDRS III in the *off*‐condition at any point during the study was more than 20 points in 1.5 years were excluded. This threshold corresponds to a more than fourfold higher change compared to previously published PPMI data^19^ and suggests alternative causes of parkinsonism.^22^

With these criteria, our analysis included 148 PwPD, which were filtered from 1710 participants from the total dataset. For eligible PwPD, we included all study visits that included a levodopa challenge in the absence of concomitant DA and levodopa treatment.

This “trial cohort” was not identical to the cohort used to calculate the predicted naïve MDS‐UPDRS III score (“MLM cohort”). Sixty‐four PwPD were part of both datasets. Baseline MDS‐UPDRS III scores were moderately different (Table S1). We, therefore, performed a sensitivity analysis using propensity score matching based on baseline MDS‐UPDRS III and disease duration. Re‐fitting the “predicted naïve MDS‐UPDRS III” model to this matched subgroup yielded a highly similar progression estimate for disease progression (2.26 points/year for the matched subgroup), supporting the robustness of the modelling approach (Table S2).

### Statistical Analyses

Statistical analyses were conducted in Python 3.10 and JASP 0.18.3. Statistical significance was set at a two‐tailed *P* < 0.05.

PwPD of the “trial cohort” were classified as “LDR observed” if they had at least one positive model‐derived LDR, that is, at least one MDS‐UPDRS III score in the practically defined *off* was smaller than the “predicted naïve” MDS‐UPDRS III score. All other PwPD were classified as “LDR absent.” To detect possible differences between the LDR observed and the LDR absent groups, categorical variables were compared using χ^2^, and continuous variables were compared using Student's *t* test.

For PwPD with observed LDR, we calculated LDR and SDR for each visit and classified visits into exactly one bin: 0 to 2, 2 to 4, 4 to 6, 6 to 8, and 8 to 10 years after levodopa initiation. No visit was assigned to more than one time interval. To detect possible changes in LDR magnitude over time, an analysis of variance (ANOVA) was conducted. Additionally, we modelled LDR proportions over time since levodopa initiation using generalized estimating equations (GEE).

LDR and SDR were correlated with demographic data and clinical scales using Pearson's correlations for normally distributed variables and Spearman's correlation for non‐normally distributed variables. For the assessments of trajectories according to LDR status, we used MLMs. As a further sensitivity analysis, we recalculated the MLMs using a stricter definition of LDR observed by requiring LDR to be at least 50% of SDR, following previous work by others.^23^ The incidence of motor fluctuations was defined as a non‐zero MDS‐UPDRS IV score and analyzed using a Kaplan–Meier analysis.

## Results

This study investigated the impact of the LDR in 148 PwPD from the PPMI dataset resembling a clinical trial testing disease modification (see Methods for inclusion and exclusion criteria). Their demographic and baseline data are displayed in Table 1.

**TABLE 1:** Demographic and clinical characteristics for the whole cohort (n = 148)

	Mean	SD	IQR
Age at diagnosis (yr)	63.9	9.7	12.5
Sex (% female)	33		
Time since diagnosis at study inclusion (yr)	0.76	0.62	0.67
Time since diagnosis until first levodopa administration (yr)	1.52	0.93	1.17
Hoehn and Yahr	1.7	0.5	1
MDS‐UPDRS I	5.4	3.5	4.0
MDS‐UPDRS II	7.6	5.1	7.25
MDS‐UPDRS III	25.6	10.7	16.25
MoCA	26.7	2.5	4.0
MAO‐B‐inhibitor treatment (n)	17		

Abbreviations: IQR, interquartile range; MDS‐UPDRS, Movement Disorders Society‐Unified Parkinson's Disease Rating Scale; MAO‐B, monoamine oxidase B; MoCA, Montreal cognitive assessment; SD, standard deviation; yr, year.

### Measuring the LDR at the Time of Levodopa Initiation

We first calculated the “measured” LDR, using the last levodopa‐naïve MDS‐UPDRS III score and the first MDS‐UPDRS III score in the practically defined *off* state after initiation of levodopa therapy. The last levodopa‐naïve MDS‐UPDRS III score was obtained up to 1.5 years before levodopa initiation (mean: 285 days, range: 61–549). Average MDS‐UPDRS III scores before initiation of levodopa were 29.1 points and 27.1 points in the practically defined *off* after initiation of levodopa (paired *t* test, *P* = 0.004). This corresponds to a mean LDR of −2 points (improvement). The average MDS‐UPDRS III score in the *on* state was 20.6 points, which corresponds to a mean SDR of −6.5‐points (paired *t* test, *P* < 0.001). A total of 85% of patients showed a positive SDR at the first visit after initiation of levodopa.

A total of 58% of PwPD showed a positive “measured” LDR (mean −7.3 points ± 5.3), that is, a lower or equal MDS‐UPDRS III *off* score after initiation of levodopa than the last pre‐levodopa MDS‐UPDRS III. This LDR represented 53% of the TR to levodopa (SDR 47%, −6.4 ± 4.7, TR: −13.8 points ± 7.1).

A total of 42% of PwPD showed a negative “measured” LDR of 5.2 ± 3.3 points, which is a higher MDS‐UPDRS III *off* score after initiation of levodopa than the last reported MDS‐UPDRS III before levodopa. This negative LDR of 5.2 points compares to a − 8.8‐point SDR in this subgroup (unpaired *t* test with *P* < 0.02 for all comparisons). PwPD with a positive “measured” LDR received a slightly higher dose of levodopa (411 mg vs. 336 mg, *t* test, *P* = 0.045).

Note that these calculations were not corrected for disease progression. Given the average delay of 40 weeks between the last MDS‐UPDRS III score before levodopa and the first MDS‐UPDRS III scores after initiation of levodopa, the “measured” LDR might underestimate the magnitude of the LDR and the proportion of patients with a positive LDR. A rapid disease progression might explain the negative LDR in some PwPD. Even with this conservative estimate, a meaningful proportion of PwPD show a LDR of relevant magnitude.

### Modelling Disease Progression and LDR Using a MLM

Next, we used a MLM to estimate “predicted naïve” MDS‐UPDRS III scores for up to 10 years after levodopa initiation. This model is based on a larger cohort of 245 PPMI participants (see Methods for details). The “predicted naïve” MDS‐UPDRS III scores were then used to calculate a “model‐derived” LDR. This approach allowed calculation of longitudinal LDR, SDR, and TR for 98 patients of the 148 PwPD included in the “trial cohort.” The other PwPD had a negative LDR at all study visits, precluding calculations of levodopa response proportions.

**FIG. 1:**
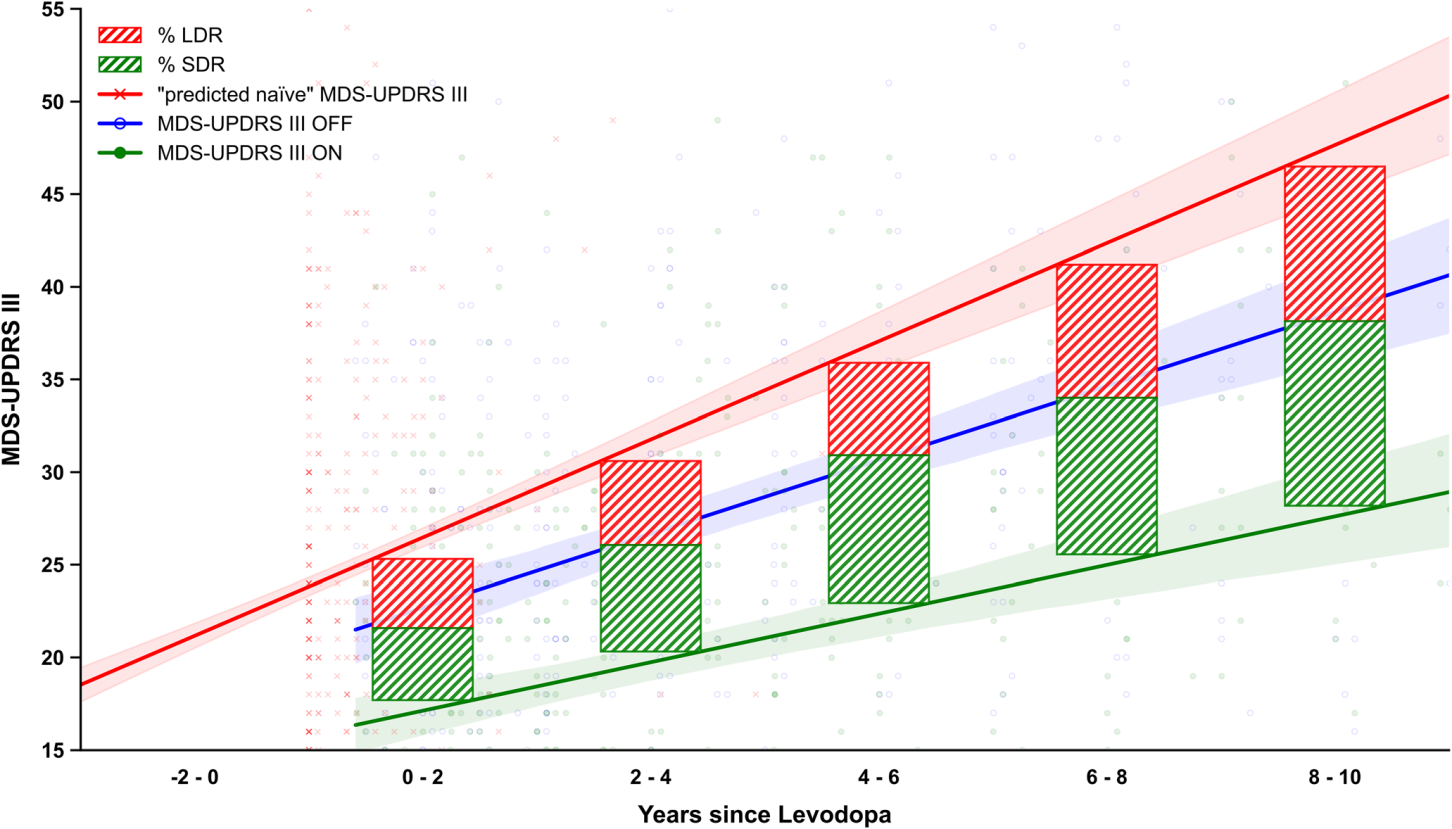
Evolution of LDR and SDR over time after the start of levodopa therapy. Boxes indicate relative magnitudes of SDR and LDR as proportions of the total response to levodopa in n = 98 participants with a positive model‐derived LDR. “Predicted naïve” MDS‐UPDRS III depicts the mixed linear model (MLM)‐based estimate derived from 245 untreated people with Parkinson's disease (PwPD) in the Parkinson's Disease Progression Marker Initiative (PPMI) cohort. LDR, long‐duration response; MDS‐UPDRS III, Movement disorders society‐Unified Parkinson's Disease Rating Scale part III; SDR, short‐duration response. [Color figure can be viewed at wileyonlinelibrary.com]

The model‐derived LDR accounted for a substantial proportion of the TR up to 10 years after the initiation of levodopa (Fig. 1 and Table 2). In fact, there was no significant difference in the magnitude of the LDR between the different time intervals (ANOVA across all abovementioned time spans with *F* = 1.74, *P* = 0.14) or over continuous time since levodopa initiation (GEE, *P* = 0.26).

**TABLE 2:** LDR in % as a proportion of the total reduction of MDS‐UPDRS III scores after levodopa initiation in participants with observed LDR (n = 98)

	0–2 yr	2–4 yr	4–6 yr	6–8 yr	8–10 yr
LDR estimate	52%	46%	39%	48%	49%
N	85	31	26	17	9

*Note*: N denotes the number of participants with available data per time interval.

Abbreviations: LDR, long‐duration levodopa response; MDS‐UPDRS, Movement Disorders Society‐Unified Parkinson's Disease Rating Scale; yr, year.

The magnitude of the model‐derived LDR only correlated with MDS‐UPDRS IV scores at a single time point, 2 to 4 years after levodopa initiation (Pearson's *r* = −0.38, *P* = 0.04, all other correlations *P* > 0.19). There were no significant correlations between the mean LDR magnitude and the last MDS‐UPDRS I, IV and Montreal Cognitive Assessment (MoCA) scores. The correlation to the last MDS‐UPDRS II score was weak (*r* = −0.20, *P* = 0.048). There was no significant correlation to the onset of fluctuations and dyskinesias, defined as a non‐zero MDS‐UPDRS IV score. There were no significant correlations between LDR magnitude and age at onset, sex, MDS‐UPDRS I or MoCA scores.

### Comparing PwPD with Observed and Absent LDR

Next, we divided PwPD into two groups according to them either having at least one positive model‐derived LDR value at any study visit (LDR observed) or only negative LDR values (LDR absent). There were no significant differences in demographic or baseline data at baseline between the LDR observed and LDR absent groups (Table 3).

**TABLE 3:** Demographic and baseline characteristics of LDR observed (n = 98) and LDR absent (n = 50) PwPD as identified by MLM‐modeling of “predicted naïve” MDS‐UPDRS III scores

Variable	LDR absent	LDR observed	*P*
n	50	98	
Age at onset in years (SD)	64.64 (9.58)	63.43 (9.72)	0.47
Sex (% female)	28%	35.7%	0.35
MDS‐UPDRS I (SD)	5.88 (3.66)	5.12 (3.43)	0.22
MDS‐UPDRS II (SD)	7.72 (5.34)	7.46 (5.08)	0.78
MDS‐UPDRS III (SD)	25.06 (10.88)	25.82 (10.71)	0.69
MDS‐UPDRS IV (SD)	–	–	
MoCA (SD)	26.70 (2.42)	26.69 (2.59)	0.99
Years since diagnosis until first levodopa administration (SD)	1.67 (0.88)	1.47 (0.96)	0.36
First levodopa dose in mg (SD)	383.30 (251.1)	380.04 (203.7)	0.93

*Note*: Student's *t* test for continuous variables, χ^2^ test for categorical variables.

Abbreviations: LDR, long‐duration levodopa response; MDS‐UPDRS, Movement Disorders Society‐Unified Parkinson's Disease Rating Scale; MLM, mixed linear model; MoCA, Montreal Cognitive Assessment; PwPD, people with Parkinson's disease; SD, standard deviation.

To assess differences in clinical progression between LDR observed and LDR absent PwPD, we fitted separate linear mixed‐effects models for several clinical outcome measures. Each model included the time since first levodopa intake, the group (LDR observed vs. LDR absent), their interaction, and the baseline score of the respective scale as fixed effects. Random intercepts were included for each subject.

For the MDS‐UPDRS III scores, we observed a significant group‐by‐time interaction (β = −1.72, *P* < 0.001), indicating a slower progression in the LDR observed group. A similar pattern emerged for motor‐related activities of daily living (MDS‐UPDRS II scores, β = −0.49, *P* < 0.001). Cognitive function declined more slowly in the LDR observed group (MoCA, β = 0.39, *P* < 0.001). No significant group difference was found for mood and behavioral symptoms (MDS‐UPDRS I; β = −0.04, *P* = 0.50). The incidence of motor fluctuations, defined as the first non‐zero MDS‐UPDRS IV score, did not differ significantly between groups (Kaplan–Meier analysis, log‐rank *P* = 0.73, incidence LDR observed: 20.9%/year, LDR absent: 16.9%/year). Yet, the magnitude of motor complications as defined by the MDS‐UPDRS IV score showed a significant group‐by‐time interaction (β = −0.16, *P* = 0.029).

Excluding the 17 PwPD treated with MAO‐B‐inhibitors as an adjunct to levodopa did not alter the distribution of LDR observed and LDR absent PwPD (χ^2^ test, *P* = 0.89 for both comparisons). Similarly, we found no influence of concomitant diabetes, cardiovascular disease, or presence of common malignant diseases on LDR status (χ^2^ test, *P* > 0.11 for all comparisons).

As a further sensitivity analysis, we used a stricter definition of LDR observed, namely LDR being at least 50% of SDR. This yielded qualitatively similar results. Only the difference in the magnitude of the MDS‐UPDRS IV score was no longer significant, consistent with the lacking difference in the Kaplan–Meier analysis described above and with the observation that the magnitude of the model‐derived LDR only correlated with MDS‐UPDRS IV scores at a single time point.

These findings suggest that a preserved model‐derived LDR is associated with slower worsening across multiple domains. Fittingly, when classifying patients into subtypes with faster and slower progression as previously described,^4^ 23% of patients in the LDR absent, but only 9% of PwPD in the LDR observed group belonged to the subtype with fast progression (χ^2^ test, *P* < 0.001). Note that only 60 PwPD in our cohort were part of the previously published analysis.

## Discussion

This study on longitudinal data of 148 PwPD from the PPMI cohort demonstrated that the LDR contributes approximately half of the TR to levodopa. LDR did not decline significantly over the course of PD. There was no robust association of the LDR with the occurrence of fluctuations or dyskinesias. Absence of a LDR was associated with faster disease progression.

Calculating a “measured” LDR showed a significant reduction of MDS‐UPDRS III scores of 2 points after levodopa was first initiated. This mean effect was similar to the finding in the ELLDOPA study after discontinuation of levodopa in the 600 mg/day levodopa‐group,^24^ but smaller than the minimal clinically important difference (minimal clinically important difference [MCID], for detecting improvement: −3.25 points, for worsening: 4.63 points).^25^ At the individual level, approximately half of PwPD in our study achieved a “measured” LDR.

Therefore, the LDR obscures MDS‐UPDRS III progression once levodopa therapy has been started in at least half of patients. Measuring MDS‐UPDRS III scores in the practically defined *off* in levodopa‐treated PwPD (and possibly also with other dopaminergic drugs^13^), therefore, underestimates disease progression in clinical trials. One may try to correct for this effect, for instance by inferring MDS‐UPDRS III progression from MLMs. Yet, it is not possible to adequately control for a confounder whose extent cannot be known.^26^ Alternatively, such trials may analyze the data of untreated patients separately, but the untreated period in such studies tends to be short, which reduces the likelihood of observing meaningful changes.^10^

The steeper increase in MDS‐UPDRS III scores in the LDR absent group may have different explanations. On the one hand, a small or absent LDR in a subpopulation of PwPD can explain a more rapid worsening of MDS‐UPDRS III scores. Conversely, rapid disease progression can explain a small or negative measured LDR. Our conservative way of defining the model‐derived LDR also underestimates the magnitude of the LDR in PwPD with rapid disease progression. Specifically, our predictions of motor progression were derived from PPMI participants in which treatment could be delayed. These participants likely exhibit a milder disease course than PwPD in which treatment needed to be initiated early.

Several additional factors might contribute to an absent LDR in a subset of PwPD. First, a minimal levodopa dose is necessary to produce a LDR and its magnitude is influenced by therapeutic regimen (eg, interdose intervals).^27^, ^28^ Indeed, PwPD without a “measured” LDR received less levodopa than those with a “measured” LDR. Second, the LDR needs many months to develop.^20^, ^21^ In some participants, the MDS‐UPDRS III assessment might have been conducted “too early” after levodopa initiation to allow development of the LDR.

More than half of PwPD in our study exhibited a “measured” LDR of 7.3 points on the MDS‐UPDRS III scale. PwPD without a “measured” LDR showed a worsening of 2.3 points up to 1.5 years after initiation of levodopa therapy. Under the assumption that the LDR is equally pronounced among all PwPD, this difference could be a statistical effect solely driven by disease progression and not by a “true” absence of LDR. Under this assumption, PwPD without an identifiable “measured” LDR would still experience a LDR of at least 7.3 points, which, when combined with the observed 2.3‐point worsening in this group, would correspond to a LDR of 10 points. Notably, the placebo group in the ELLDOPA trial worsened by 7.8 ± 9.0 points on the UPDRS III scale after 40 weeks.^24^ Hence, the heterogeneity in disease trajectories makes it impossible to infer from our data whether the absence of a LDR in our cohort is a statistical effect mediated by disease progression or whether this is part of the heterogeneity of PD itself.

Incorporating the considerations made above, we argue that a lack of a (statistically) identifiable LDR identifies PwPD with faster disease progression, which may help to reduce the number of PwPD that need to be included into clinical trials of disease‐modifying drugs. This approach would be practical, as it only relies on two MDS‐UPDRS III scores, one before the initiation of levodopa and one in the practically defined *off*. If patients have a higher UPDRS III score in the practically defined *off* state approximately 1 year after the initiation of levodopa, their rate of disease progression is likely in the faster half.

With respect to motor fluctuations, our data suggest that the magnitude of the LDR remains stable over the course of PD, which is consistent with work by others.^8^, ^14^, ^29^ We argue that a relative shift of the proportions of LDR and SDR is unlikely to be the primary substrate for the occurrence of motor fluctuations.^14^, ^30^ Accordingly, we observed no association of LDR status with the onset of motor fluctuations and the association between LDR status, and the magnitude of motor fluctuations was not robust when a stricter definition of the LDR was used. Still, the number of PwPD in our study with LDR data beyond 6 years after levodopa initiation is low and the stability of LDR magnitude over time might in part be explained by attrition bias.

**FIG. 2:**
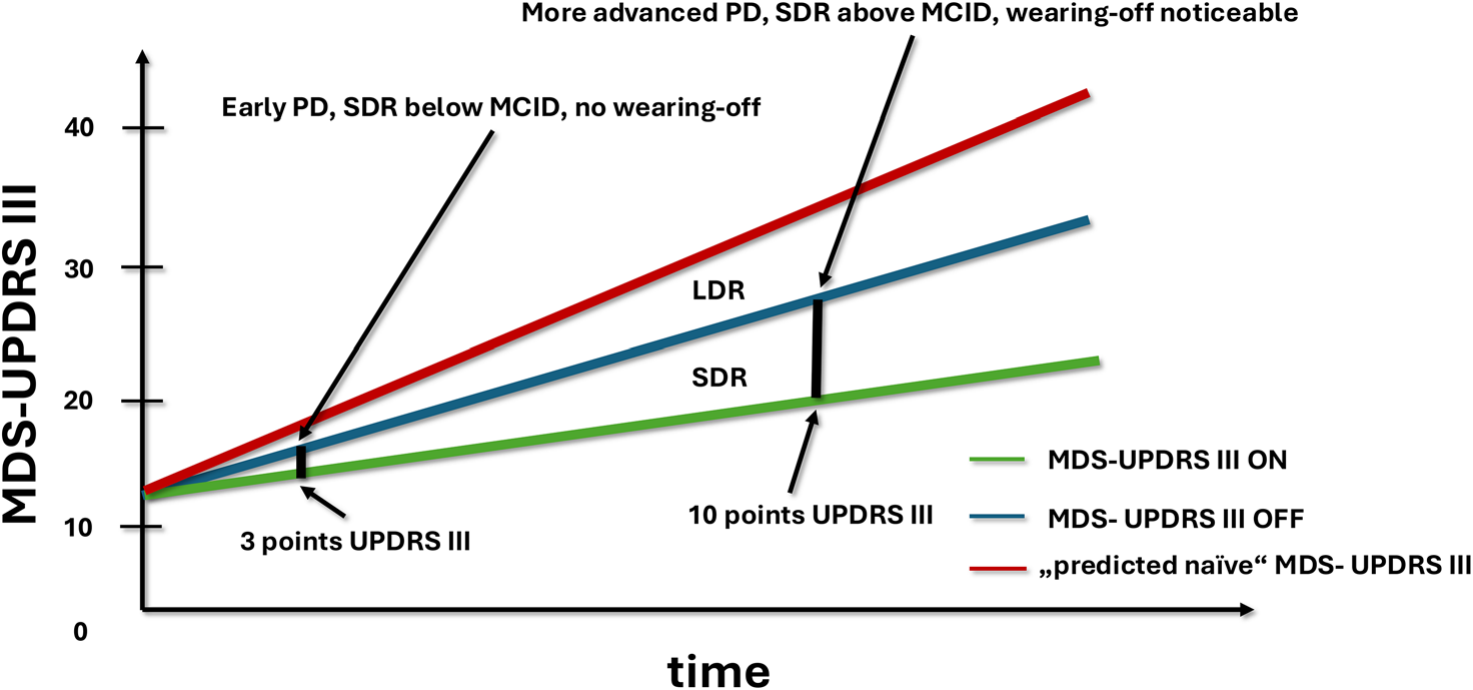
Proposed mechanism for the clinical occurrence of wearing‐off phenomena in PD. LDR, long‐duration response; MCID, minimally clinically important difference; MDS‐UPDRS III, Movement Disorders Society‐Unified Parkinson's Disease Rating Scale part III; PD, Parkinson's disease; SDR, short‐duration response. [Color figure can be viewed at wileyonlinelibrary.com]

The absolute extent of the SDR may better account for the occurrence of wearing‐off phenomena as motor disability progresses. In line with observations made by others,^31^ we hypothesize that wearing‐off becomes noticeable as soon as the SDR crosses the threshold to the MCID (Fig. 2). This would be in line with the observation that wearing‐off can be measured in early PD and is underdiagnosed in clinical practice.^32^ The occurrence of dyskinesias, on the other hand, has been causally linked to degenerative changes to the striatum as PD progresses.^33^ Consequently, the occurrence of dyskinesias is closely linked to disease duration.^34^ As such, very early PwPD are unable to develop dyskinesias and will experience wearing‐off only to an amount where it is not bothersome. It seems plausible that both phenomena are not causally linked (ie, not caused by levodopa therapy), but tend to occur together, as the underlying progression of PD causes both, but wearing‐off is more closely linked to pharmacokinetic aspects of levodopa therapy, while dyskinesias are caused by striatal changes.

Overall, these findings highlight the clinical relevance of the LDR in PD. LDR contributes substantially to the TR to levodopa in a majority of PwPD and remains stable over the course of the disease. It obscures the interpretation of MDS‐UPDRS III progression in treated patients and challenges the use of this measure as a surrogate for disease progression in clinical trials. Our data further suggest that a loss of LDR is not the primary substrate of motor fluctuations or dyskinesias.

## Author Roles

(1) Research project: A. Conception, B. Execution; (2) Statistical Analysis: A. Design, B. Execution, C. Review and Critique; (3) Manuscript Preparation: A. Writing of the First Draft, B. Review and Critique.

N.S.: 1A, 1B, 2A, 2B, 3A

T.F.: 1A, 1B, 2A, 2B, 2C, 3B

T.H.: 2C, 3B

B.F.: 2C, 3B

## Financial Disclosures of All Authors (for the Past 12 Months)

N.S. reports a travel grant by the Parkinson's Foundation. T.F. and T.H. have nothing to report. B.F. has received advisory and consultancy honoraria from AbbVie, Bial, and is an investigator on studies funded by AbbVie, Biogen, CHDI, Denali, DFG, The Michael J. Fox Foundation for Parkinson's Research, Roche and Teva.

## Supporting information


**Figure S1.** Schematic depiction of differential levodopa effects. LDR, long‐duration response; SDR, short‐duration response; TR, total response.

**Table S1.** Comparison of baseline characteristics between untreated PPMI participants used to estimate predicted drug‐naïve motor progression (“MLM cohort”) and the cohort simulating a clinical trial that was used for analysis of the LDR (“trial cohort”). MDS‐UPDRS, movement disorders society‐sponsored revision of the Unified Parkinson's disease rating scale; MoCA, Montreal cognitive assessment; SD, standard deviation.
**Table S2.** Comparison of mixed linear model (MLM) estimates of MDS‐UPDRS III progression between the full MLM cohort and an MLM sub‐cohort matched to the “trial cohort” using propensity score matching based on disease duration and MDS‐UPDRS III baseline scores. Slope denotes the estimated annual increase in MDS‐UPDRS III scores. MAE, mean absolute error; MDS‐UPDRS, movement disorders society‐sponsored revision of the Unified Parkinson's disease rating scale; RMSE, root mean squared error.

## Data Availability

The data that support the findings of this study are available from the corresponding author upon reasonable request.

## References

[1] Greenland JC , Williams‐Gray CH , Barker RA . The clinical heterogeneity of Parkinson's disease and its therapeutic implications. Eur J Neurosci 2018;49(3):328–338. 10.1111/ejn.14094 30059179

[2] Höglinger G , Schulte C , Jost WH , et al. GBA‐associated PD: chances and obstacles for targeted treatment strategies. J Neural Transm 2022;129(9):1219–1233. 10.1007/s00702-022-02511-7 35639160 PMC9463270

[3] Chen Q , Scherbaum R , Gold R , Pitarokoili K , Mosig A , Zella S , Tönges L . Data‐driven subtyping of Parkinson's disease: comparison of current methodologies and application to the Bochum PNS cohort. J Neural Transm 2023;130(6):763–776. 10.1007/s00702-023-02627-4 37000269 PMC10199871

[4] Hähnel T , Raschka T , Sapienza S , et al. Progression subtypes in Parkinson's disease identified by a data‐driven multi cohort analysis. NPJ Parkinsons Dis 2024;10(1):1–13. 10.1038/s41531-024-00712-3 38698004 PMC11066039

[5] Dadu A , Satone V , Kaur R , et al. Identification and prediction of Parkinson's disease subtypes and progression using machine learning in two cohorts. NPJ Parkinsons Dis 2022;8(1):1–12. 10.1038/s41531-022-00439-z 36526647 PMC9758217

[6] Hutchison RM , Fraser K , Yang M , et al. Cinpanemab in early Parkinson disease: evaluation of biomarker results from the phase 2 SPARK clinical trial. Neurology 2024;102(5):e209137. 10.1212/WNL.0000000000209137 38315945

[7] Eijsvogel P , Misra P , Concha‐Marambio L , et al. Target engagement and immunogenicity of an active immunotherapeutic targeting pathological *α*‐synuclein: a phase 1 placebo‐controlled trial. Nat Med 2024;30:1–10. 10.1038/s41591-024-03101-8 38902546 PMC11405261

[8] Nagao K , Ding C , Ganga G , et al. Inferring the long duration response to levodopa in Parkinson's disease. Parkinsonism Relat Disord 2019;60:133–137. 10.1016/j.parkreldis.2018.09.002 30217541

[9] Pagano G , Boess FG , Taylor KI , et al. A phase II study to evaluate the safety and efficacy of Prasinezumab in early Parkinson's disease (PASADENA): rationale, design, and baseline data. Front Neurol 2021;12:705407. 10.3389/fneur.2021.705407 34659081 PMC8518716

[10] Pagano G , Taylor KI , Anzures‐Cabrera J , et al. Trial of Prasinezumab in early‐stage Parkinson's disease. N Engl J Med 2022;387(5):421–432. 10.1056/NEJMoa2202867 35921451

[11] Nikolcheva T , Pagano G , Pross N , et al. A phase 2b, multicenter, randomized, double‐blind, placebo‐controlled study to evaluate the efficacy and safety of intravenous prasinezumab in early‐stage Parkinson's disease (PADOVA): rationale, design, and baseline data. Parkinsonism Relat Disord 2025;132:107257. 10.1016/j.parkreldis.2024.107257 39798255

[12] Cotzias GC , Van Woert MH , Schiffer LM . Aromatic amino acids and modification of parkinsonism. N Engl J Med 1967;276(7):374–379. 10.1056/NEJM196702162760703 5334614

[13] Anderson E , Nutt J . The long‐duration response to levodopa: phenomenology, potential mechanisms and clinical implications. Parkinsonism Relat Disord 2011;17(8):587–592. 10.1016/j.parkreldis.2011.03.014 21530356

[14] Cilia R , Cereda E , Akpalu A , et al. Natural history of motor symptoms in Parkinson's disease and the long‐duration response to levodopa. Brain 2020;143(8):2490–2501. 10.1093/brain/awaa181 32844196 PMC7566883

[15] Brissenden JA , Scerbak T , Albin RL , Lee TG . Motivational vigor in Parkinson's disease requires the short and long duration response to levodopa. Mov Disord 2024;39(1):76–84. 10.1002/mds.29659 38062630 PMC10842158

[16] Evers LJW , Krijthe JH , Meinders MJ , Bloem BR , Heskes TM . Measuring Parkinson's disease over time: the real‐world within‐subject reliability of the MDS‐UPDRS. Mov Disord 2019;34(10):1480–1487. 10.1002/mds.27790 31291488 PMC6851993

[17] Nutt JG , Carter JH , Lea ES , Sexton GJ . Evolution of the response to levodopa during the first 4 years of therapy. Ann Neurol 2002;51(6):686–693. 10.1002/ana.10189 12112073

[18] PPMI Protocol; https://www.ppmi-info.org/study-design/research-documents-and-sops.

[19] Holden SK , Finseth T , Sillau SH , Berman BD . Progression of MDS‐UPDRS scores over five years in De novo Parkinson disease from the Parkinson's progression markers initiative cohort. Mov Disord Clin Pract 2018;5(1):47–53. 10.1002/mdc3.12553 29662921 PMC5898442

[20] Pauwels A , Phan ALG , Ding C , Phan TG , Kempster PA . Rate of motor progression in Parkinson's disease: a systematic review and meta‐analysis. Front Neurol 2024;15:1452741. 10.3389/fneur.2024.1452741 39391167 PMC11464440

[21] Kempster PA . Understanding the progression of Parkinson's disease: a review. BMJ Neurol Open 2025;7(2):e001215. 10.1136/bmjno-2025-001215 PMC12421158 40937041

[22] Seppi K , Yekhlef F , Diem A , et al. Progression of parkinsonism in multiple system atrophy. J Neurol 2005;252(1):91–96. 10.1007/s00415-005-0617-2 15654560

[23] Sciacca G , Mostile G , Disilvestro I , Donzuso G , Nicoletti A , Zappia M . Long‐duration response to levodopa, motor learning, and neuroplasticity in early Parkinson's disease. Mov Disord 2023;38(4):626–635. 10.1002/mds.29344 36840442

[24] Levodopa and the progression of Parkinson's disease. N Engl J Med 2004;351(24):2498–2508. 10.1056/NEJMoa033447 15590952

[25] Horváth K , Aschermann Z , Ács P , et al. Minimal clinically important difference on the motor examination part of MDS‐UPDRS. Parkinsonism Relat Disord 2015;21(12):1421–1426. 10.1016/j.parkreldis.2015.10.006 26578041

[26] Fewell Z , Davey Smith G , Sterne JAC . The impact of residual and unmeasured confounding in epidemiologic studies: a simulation study. Am J Epidemiol 2007;166(6):646–655. 10.1093/aje/kwm165 17615092

[27] Zappia M , Oliveri RL , Bosco D , et al. The long‐duration response to l‐dopa in the treatment of early PD. Neurology 2000;54(10):1910–1915. 10.1212/WNL.54.10.1910 10822428

[28] Zappia M , Oliveri RL , Montesanti R , et al. Loss of long‐duration response to levodopa over time in PD: implications for wearing‐off. Neurology 1999;52(4):763–767. 10.1212/wnl.52.4.763 10078724

[29] Wider C , Russmann H , Villemure JG , Robert B , Bogousslavsky J , Burkhard PR , Vingerhoets FJG . Long‐duration response to levodopa in patients with advanced Parkinson disease treated with subthalamic Deep brain stimulation. Arch Neurol 2006;63(7):951. 10.1001/archneur.63.7.951 16831963

[30] Poewe W , Espay AJ . Long duration response in Parkinson's disease: levodopa revisited. Brain 2020;143(8):2332–2335. 10.1093/brain/awaa226 32844192

[31] Nutt JG . Pharmacokinetics and pharmacodynamics of levodopa. Mov Disord 2008;23(S3):S580–S584. 10.1002/mds.22037 18781675

[32] Stocchi F , Antonini A , Barone P , et al. Early DEtection of wEaring off in Parkinson disease: the DEEP study. Parkinsonism Relat Disord 2014;20(2):204–211. 10.1016/j.parkreldis.2013.10.027 24275586

[33] Espay AJ , Morgante F , Merola A , et al. Levodopa‐induced dyskinesia in Parkinson disease: current and evolving concepts. Ann Neurol 2018;84(6):797–811. 10.1002/ana.25364 30357892

[34] Cilia R , Akpalu A , Sarfo FS , et al. The modern pre‐levodopa era of Parkinson's disease: insights into motor complications from sub‐Saharan Africa. Brain 2014;137(10):2731–2742. 10.1093/brain/awu195 25034897 PMC4163032

