# Supplementary material for: Long‐Duration Response to Levodopa in the PPMI‐Cohort
Source: Mov Disord. 2026 May 9;41(8):2033–41. doi: 10.1002/mds.70344 (PMC13518283; doi:10.1002/mds.70344)
Supplement: Supplementary file 1 [file MDS-41-2033-s001.docx]

**Supplementary Tables**

***Supplementary Table 1.*** Comparison of baseline characteristics between untreated PPMI participants used to estimate predicted drug-naïve motor progression (“MLM cohort”) and the cohort simulating a clinical trial that was used for analysis of the LDR (“trial cohort”). MDS-UPDRS: Movement Disorders Society-Sponsored Revision of the Unified Parkinson’s Disease Rating Scale, MoCA: Montreal Cognitive Assessment, SD: standard deviation.

| **Variable** | **MLM cohort** | **trial cohort** | **p** |
| --- | --- | --- | --- |
| **N** | 245 | 148 |  |
| **Age at onset in years (SD)** | 62.7 (9.58) | 63.9 (9.7) | 0.27 |
| **Sex (% female)** | 33% | 33% | 1 |
| **MDS-UPDRS I (SD)** | 4.4 (3.66) | 5.4 (3.5) | 0.005 |
| **MDS-UPDRS II (SD)** | 6.0 (5.34) | 7.6 (5.2) | 0.003 |
| **MDS-UPDRS III (SD)** | 21.8 (10.88) | 25.6 (10.7) | <0.001 |
| **MDS-UPDRS IV (SD)** | – | – |  |
| **MoCA (SD)** | 27.0 (2.3) | 26.7 (2.5) | 0.13 |

**Supplementary Table 2**. Comparison of mixed linear model (MLM) estimates of MDS-UPDRS III progression between the full MLM cohort and an MLM sub-cohort matched to the “trial cohort” using propensity score matching based on disease duration and MDS-UPDRS III baseline scores. Slope denotes the estimated annual increase in MDS-UPDRS III scores. MAE: mean absolute error, MDS-UPDRS: Movement Disorders Society-Sponsored Revision of the Unified Parkinson’s Disease Rating Scale, RMSE: root mean squared error.

| **Model** | **N** | **Observations** | **Slope** | **MAE** | **RMSE** |
| --- | --- | --- | --- | --- | --- |
| **MLM cohort** | 245 | 1155 | 2.65 | 3.87 | 5.27 |
| **Propensity-matched MLM sub-cohort** | 147 | 730 | 2.26 | 3.92 | 5.36 |
